# Using the Extended Acceptance Model to Understand Continuance Intention of Dockless Bike-Sharing

**DOI:** 10.3389/fpsyg.2022.786693

**Published:** 2022-02-07

**Authors:** Xiadi Li, Hanchuan Lin

**Affiliations:** ^1^School of Management, Zhejiang University of Technology, Hangzhou, China; ^2^China Institute for Small and Medium Enterprise, Zhejiang University of Technology, Hangzhou, China

**Keywords:** dockless bike-sharing, continuance intention, environmental concern, descriptive social norms, injunctive social norms

## Abstract

Despite the fact that dockless bike-sharing (DBS) usage first experienced explosive growth, its continuous usage rate remains low. The ultimate success of a DBS service is more dependent on its continued usage rather than its initial adoption. Following the extended technology acceptance model (TAM), this study aims to explore factors that influence the continuance intention of DBS users. The framework of research was validated using a sample of 369 DBS users in China. The results show that perceived usefulness and perceived ease of use positively influence a user’s intention to continue using DBS. Both descriptive social norms and injunctive social norms are positively related to the continuance intention of DBS users. Moreover, environmental concern significantly affects the continuance intention of a user indirectly *via* perceived usefulness and perceived ease of use. Furthermore, the extended TAM has stronger prediction ability than the original TAM in the context of DBS services.

## Introduction

Fueled by advances in mobile technology, the Internet of Things (IoT), and growing public awareness on environmental issues, bike-sharing has become a form of public transportation (PT) in many cities worldwide (Chen et al., 2020). In 2016, a new generation of bike-sharing, namely dockless bike-sharing (DBS), emerged in China and became very popular (Manca et al., 2019). DBS can be defined as a bicycle-renting service provided by private firms without fixed stations (Gu et al., 2019). Compared with traditional bike-sharing services where a user needs to borrow a bike from a docking station and return it to another docking station within the same system, DBS services allow users to locate shared bikes anywhere parking is permitted. The main advantage of DBS is its convenience, as locating, unlocking, dropping, and paying for the use of a bike is primarily completed through a smartphone application (Chen et al., 2020). Moreover, DBS is a supplement to PT, and it can effectively ease the travel problem of “the last miles” in daily commuting of people (Wei et al., 2021). The development of DBS potentially contributes to reduction in vehicle trips and, thus, enhances the possibility of green travel. For example, Ma et al. (2019) found that one unit increment of DBS increases bus ridership by approximately 50% during morning peak time on weekdays. Hence, DBS services are supported by governments and are considered to be an indispensable part of long-term urban green transportation systems.

However, despite the growing convenience of using bike-sharing services and environmental benefits of DBS, the report written by Jiang et al. (2020) demonstrates that only 61% of users have been using DBS for more than 12 months, which indicates that some registered users are inactive, and that the continued usage rate of the system is not high. Although initial adoption is necessary for DBS operators, successful diffusion of DBS services depends on the continued interest of users (Bhattacherjee, 2001a). Therefore, this study aims to explore factors that affect the continuance intention of DBS users to help DBS service operators retain their customers. In this article, continuance intention of DBS users refers to regular long-term use of DBS services by users (Kim and Kang, 2016; Wang et al., 2019).

Because DBS is a new mode of transportation, its promotion is a topic that has attracted the interest of scholars. In academia, existing studies have investigated continuance intention of DBS users either from the perspective of service quality (Shao et al., 2020; Zhang et al., 2020) or from the perspective of sustainable motivation (Chen, 2016a). However, very little research has combined technological and environmental factors within a single theoretical framework to explain DBS continuance intention. In general, the DBS program has two distinguishing characteristics. First, the usage of DBS is beneficial to environmental protection, as it promotes green low carbon behaviors. Second, it is an innovative model of transportation, because its operation is supported by information technology. Hence, DBS users are influenced by technological and environmental factors simultaneously. Because the popularity of the DBS service is mainly driven by its use of advanced technology, this study aims to utilize the technology acceptance model (TAM), which has been verified to successfully predict the continuance behaviors of various information system (IS) applications. Two critical factors in the TAM, perceived usefulness and perceived ease of use, represent the benefits that technological advancement of DBS brings. These two constructs might change after the first adoption, and changes might influence continuance intention. Therefore, the TAM is applicable to explore post-adoption behavior.

This study is one of the efforts to extend the scope to discover the mechanism behind users’ continuance usage of transportation technologies (e.g., Sun and Duan, 2019; Nguyen-Phuoc et al., 2020). Specifically, it enhances our understanding of DBS usage and contributes to the development of the TAM in two ways. First, TAM predictors emphasize the influence of generic attributes of an information system, such as usefulness and ease of use, but factors that trigger perceived usefulness and perceived ease of use remain unclear in the context of DBS (Wu R. et al., 2019). To tackle this limitation, the article extends the TAM from a green transportation technology perspective and explores whether environmental concern would influence user perceptions toward DBS and, in turn, encourage future use. In addition, the article elaborates the role of two-dimensional social norms with regard to the TAM when predicting continuance usage of DBS. Even though social norms have been known to influence the initial adoption of DBS, few studies have explored the influence of social norms on the post-adoption of DBS, especially with respect to different roles of descriptive norms and injunctive norms. Descriptive social norms refer to what is typically done within a group, and injunctive social norms refer to whether a certain behavior is normally encouraged by a certain group (Cialdini and Goldstein, 2004). Thus, it is essential to uncover different impacts of descriptive social norms and injunctive social norms on the continuance intention of DBS users. To fill the gap identified above, this study aims to investigate DBS continuance behavior from an integrated perspective by simultaneously considering the influence of technological, environmental, and socio-psychological factors.

The contributions of this study are as follows: first, it is among the first attempts to explain post-adoption behaviors of DBS users by applying the extended TAM that combines technological, social-psychological, and environmental factors. Second, this study identifies the role of environmental concern as an important stimulus that can motivate perceived usefulness and perceived ease of use in the context of DBS. Perceived usefulness is an outcome of environmental concern, descriptive social norms, and injunctive social norms, while perceived ease of use is influenced by environmental concern. Third, the article further verifies different roles that descriptive social norms and injunctive social norms play in motivating the continuance intention of DBS users. Finally, based on the results, this article offers new insights into the promotion of DBS continuance behavior that DBS operators and governments could find useful.

The remainder of the article is organized as follows: Section “LITERATURE REVIEW AND RESEARCH HYPOTHESES” presents the associated theory, factors that affect the continuance intention of DBS users, and the proposed research model. Then, the article explains how data and research sample were collected, followed by a description of the results. Finally, the closing sections discuss the results and propose relevant implications and further recommendations for studies in this research area.

## Literature Review and Research Hypotheses

Dockless bike-sharing (DBS) continuance intention can be defined as the continued usage intention of DBS by adopters, where a post-adoption decision follows an initial adoption choice (Bhattacherjee, 2001a). Previous studies have primarily explored the continuance intention of DBS users from two perspectives. The mainstream focus involves treating DBS as a new information service and explores whether the new technology improves service quality and usage experience, and thus enhances the continuance intention of DBS. For example, scholars have verified that there is a positive relationship among perceived service quality, satisfaction, and continuance intention of DBS users through the application of a quality-loyalty model (Zhou and Zhang, 2019; Shao et al., 2020). Additionally, two TAM constructs (perceived usefulness and perceived ease of use) were found to be important predictors of intention to continue using DBS (Cheng et al., 2019; Wu R. et al., 2019; Kim and Kim, 2020). The other stream explores the continuance intention of DBS users from a pro-environmental perspective. For instance, Chen (2016b) elucidated important roles that perceived green usefulness, perceived green value, and pro-environmental attitudes play in the continued usage behavior of DBS users; however, both streams failed to explain DBS continuance behavior from an integrated perspective. As the nature of DBS is both instrumental and environmental, the continuance intention of users is jointly affected by rational and irrational factors (Huang et al., 2019). Moreover, Manca et al. (2019) have identified that individual travel choices are affected by irrational factors as well as rational factors, because people are constantly influenced by thoughts and behaviors of others.

According to previous studies, technological, environmental, and socio-psychological factors are all vital elements that help explain users’ continuance intention of green transportation services with new technology (Chen, 2016a; Cheng et al., 2019; Chen et al., 2020). As the development of DBS is mainly driven by mobile technology advancement, this study aims to develop a comprehensive model based on the TAM (Davis, 1989), as the TAM has been successfully used to explain the continuance intention of information services in various contexts (Wang, 2014; Yuan et al., 2016; Huang and Ren, 2020). Moreover, previous studies have identified that the predictive power of the TAM could be improved by incorporating extra external factors (Hu et al., 1999; Weng et al., 2017). Hence, environmental concern and two-dimensional social influence are incorporated into the TAM for the following reasons: first, given the potential positive impact that DBS services could have on the environment and long-lasting effect of environmental concern on human behaviors (Chen and Lu, 2016; Wang et al., 2018), the role of environmental concern in increasing the continuance intention of DBS users merits attention. Additionally, scholars have increasingly emphasized the importance of differentiating descriptive social norms and injunctive social norms in explaining human behaviors, as they have different effects (Rivis and Sheeran, 2003; Cialdini and Goldstein, 2004; Beldad and Hegner, 2018). However, there has been limited research that explores their different impacts, so this article incorporates both descriptive social norms and injunctive social norms to demonstrate their different influences. Hence, this study aims to develop a comprehensive model by integrating environmental concern, descriptive social norms, and injunctive social norms into the TAM, and identifying how these factors affect the continuance intention of DBS users.

### Technology Acceptance Model

The TAM, developed by Davis (1989), assumes that perceived usefulness and perceived ease of use contribute to a user’s adoption of one specific technology. Perceived usefulness refers to the extent to which new technology could improve the work productivity of a person, and perceived ease of use refers to the extent to which a specific new technology could be used without much effort (Davis, 1989). By conducting a meta-analysis, King and He (2006) showed that these two variables explain almost 50% of the variance in information technology or information system usage intention. Although the TAM was originally used to examine initial acceptance, scholars have recently started to apply this model to predict continuance behavior and post-adoption behavior and, thus, have validated its predictive power in various technological contexts, including transportation technology (Lin and Filieri, 2015; Weng et al., 2017). For instance, Lin and Filieri (2015) confirmed the validity of the TAM in the context of online transportation services by demonstrating that perceived usefulness and perceived ease of use jointly predict the intention of users to continue using online check-in services. In the context of bike-sharing, a few recent studies have adopted the TAM as a theoretical model to predict the initial adoption intention of an individual toward bike-sharing (Hazen et al., 2015; Chen and Lu, 2016; Wu R. et al., 2019).

Because DBS is a new technology, it is appropriate to explore the continuance intention of users by adopting the TAM as a basic theoretical model. In this study, perceived usefulness refers to the degree to which users believe that DBS could make their trip efficient. Perceived ease of use is defined as the degree to which a user accepts that continued use of DBS will be free of effort. In general, users are inclined to keep using a new technology that could make their life easier and more efficient. Once individuals have positive perceptions of DBS and view it as an efficient means of transportation, they are more likely to continue using it. It is also necessary for users to feel that this new generation of bike-sharing is easy to use, so that they are willing to use it regularly. Furthermore, when the service is perceived as effortless to use, users are more likely to think that it is helpful (Weng et al., 2017). Empirical research studies have confirmed that perceived ease of use can reinforce users’ perceived usefulness (Cheng and Huang, 2013; Mohammadi, 2015). Hence, the research proposes the following hypotheses:

H1: Perceived usefulness is positively related to the continuance intention of DBS users.

H2: Perceived ease of use is positively related to the continuance intention of DBS users.

H3: Perceived ease of use among DBS users is positively related to their perceived usefulness of DBS.

### Social Influence: Descriptive and Injunctive Social Norms

Social influence can be defined as the pressure that an individual experiences from important referents or social networks (Ajzen, 1991; Yang et al., 2012). Previous research has found that social influence plays a critical role in promoting technology usage behaviors and pro-environmental behaviors (Young, 2009; Ru et al., 2018), but most studies measured it as conceptually similar to the subjective norm in the theory of planned behavior (TPB) (Jansson et al., 2017; Yang and Zeng, 2018). Scholars have realized that social influence should be classified into two parts, injunctive social norms and descriptive social norms (Ajzen, 1991; Cialdini and Goldstein, 2004), which represent different aspects of external normative influence. Injunctive social norms in this study are defined as the belief of a user about whether others consider continuance usage of DBS is appropriate or inappropriate. An individual is more likely to engage in a specific behavior if they perceive that others expect them to act in a certain way (Ru et al., 2018). Hence, expectations that other people have about DBS continuance behaviors may affect an individual’s intention to continue using DBS. Descriptive social norms in this study refer to continuance usage behaviors of DBS displayed by important others (Schultz et al., 2008). People are uncomfortable with uncertainty, so they would seek to reduce their perceived level of risk by following ideas and decisions of others (Jansson et al., 2017). In the DBS scenario, risk perception refers to the perception of loss of personal information, as the use of DBS is achieved through the use of an app. Therefore, if people find important others continue using DBS, they will be more convinced of the utility of DBS in daily traveling and become more trusting of DBS operators. Additionally, previous studies have found that descriptive social norms and injunctive social norms positively affect post-adoption behaviors, such as loyalty toward green modes of travel (Doran and Larsen, 2016), towel reuse intention in a hotel (Han and Hyun, 2018), and fitness app continuous use behavior (Beldad and Hegner, 2018). Hence, based on previous studies, it can be inferred that people tend to follow expectations and actual behaviors of important others when deciding whether to continue conducting a specific behavior (Ru et al., 2018). In the context of this research, when users feel that important friends, colleagues, and family members think that they should continue using DBS or personally witness their continuous use of DBS, they will feel pressure and be more likely to continue using DBS in the long run. Thus, this research proposed the following hypotheses:

H4: Descriptive social norms are positively related to the continuance intention of DBS users.

H5: Injunctive social norms are positively related to the continuance intention of DBS users.

Several studies have also found that descriptive social norms and injunctive social norms can influence continuance intention indirectly by perceived usefulness (Venkatesh and Davis, 2000; Beldad and Hegner, 2018). For example, in the context of fitness app usage, when important others use a certain app, it provides an individual with strong cues to believe in the value of that product, and if important others expect to use a specific app continuously, it has a positive impact on a user’s assessment of that application (Beldad and Hegner, 2018). Similarly, descriptive social norms and injunctive social norms could enhance the belief of an individual in the usefulness of DBS services in daily travel. When important others display a positive evaluation of DBS and continually use it, users will feel an enhanced sense of confidence and be convinced of the usefulness of DBS. Hence, this study proposes the following hypotheses:

H6: Descriptive social norms are positively related to users’ perceived usefulness of DBS.

H7: Injunctive social norms are positively related to users’ perceived usefulness of DBS.

### Environmental Concern

Environmental concern refers to the extent to which consumers are conscious of environmental problems and make contributions to fix them (Lee et al., 2014; Ariffin et al., 2016). With the deterioration of the ecosystem, humans have to face increasingly serious environmental issues and are more inclined to adopt pro-environmental behaviors to protect the environment (Cheng et al., 2019). In the context of green marketing, environmental concern is considered to be a powerful predictor of pro-environmental behaviors (Wu J. et al., 2019; Zhang et al., 2020). Zhang et al. (2020) emphasized the important role that environmental concern plays in influencing low-carbon behaviors in the post-adoption stage. Individuals who have stronger environmental concerns are more likely to maintain pro-environmental behaviors (Ha and Janda, 2012; Emekci, 2019). In this context, users make contributions to decrease carbon dioxide emission and protect the environment through their continuous use of DBS.

Previous studies have confirmed that environmental concern indirectly influences pro-environmental behaviors *via* other variables (Verma et al., 2019; Wu J. et al., 2019). Several studies have revealed that individuals with higher environmental concerns are more likely to hold a positive attitude toward environment-friendly products and services, which then results in consumption intention (Jaiswal and Kant, 2018; Verma et al., 2019). For example, Wu J. et al. (2019) have confirmed that environmental concern exerts a positive indirect effect on autonomous vehicle (AV) adoption *via* perceived ease of use and perceived green usefulness. In this study, perceived usefulness and perceived ease of use are variables that represent users’ cognitive evaluation of DBS systems. People with higher environmental concern are more inclined to form positive evaluations of DBS systems, mitigate negative evaluations of DBS systems, and regard DBS as a practical alternative mode of travel that is easy to use. Consequently, the anxiety of users about the environment will be eased by the continuous usage of DBS. Hence, the following hypotheses are proposed:

H8: Environmental concern is positively related to users’ perceived usefulness of DBS.

H9: Environmental concern is positively related to users’ perceived ease of use of DBS.

**FIGURE 1:**
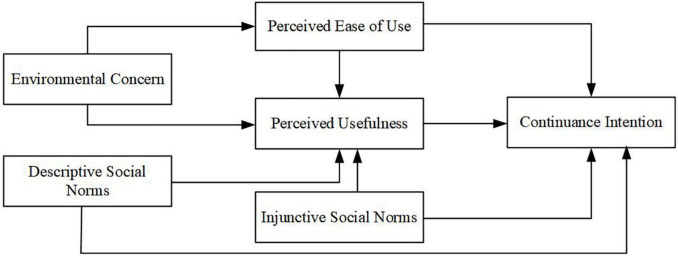
Conceptual framework.

The conceptual framework based on the proposed hypotheses is presented in Figure 1.

## Research Methodology

To examine the research model and test the hypotheses empirically, this study needed to collect data on the variables. Therefore, an on-site survey was conducted in Hangzhou, a first-tier city in China that has a large population of DBS users. Considering its population density, number of DBS users, traffic levels, vehicle use, and transportation mode, we can infer that the usage of DBS in other cities that launched DBS programs is similar to that of Hangzhou. Hence, Hangzhou was selected as the target city in this study. Information on measurements and data collection is described below.

### Measurements

Dimensions of the conceptual model include continuance intention, perceived usefulness, perceived ease of use, descriptive social norms, injunctive social norms, and environmental concern. All measurement items in this study were adopted from previous studies to ensure the validity of construct contents. Appropriate amendments were made to fit the context of DBS services in China. All items used a 7-point Likert scale from 1 (strongly disagree) to 7 (strongly agree). The questionnaire was initially created in English. With the assistance of several bilingual translators from a college, the English version of the items was then translated into Chinese following the back-translation process. Next, a focus group consisting of two professors and three postgraduates checked that all the measurements were easy to understand. After the first revision of the questionnaire, 30 college students who had used DBS in the previous month were invited to take part in the preliminary study to test the validity of the instruments. By conducting factor analysis, the results showed that all the items were loaded on their expected variables. The final questionnaire is presented in Appendix 1.

### Sample and Data Collection

In this research, the sample frame consisted of actual users of DBS (e.g., Mobike or Hellobike) in Hangzhou, China. The main reason for selecting users from Hangzhou is that Hangzhou has a long history of bike-sharing usage, and it has a large number of actual DBS users (Shaheen et al., 2011; Chen et al., 2020). The data were collected through an on-site survey from August 3 to October 31, 2020. Three investigators were asked to work from 5:00 p.m. to 7:00 p.m. on two weekdays and from 3:00 p.m. to 6:00 p.m. on one day of the weekend, as this period is the peak time of using DBS. The investigators were allowed to randomly select three days each week to conduct the investigations according to their schedules. Each investigator was asked to collect at least 150 samples before the end of October, and to continue collecting questionnaires until they reached the target number. Face-to-face questionnaires were randomly distributed to people who were waiting at metro and bus stations and people who were sitting in cafes around commercial areas. The reason for choosing metro and bus stations is that nearly 91% of respondents used DBS to connect to public transport (Jiang et al., 2020).

The target group for this study consisted of users who had ridden DBS in the last month. Questionnaires were mainly collected from bus stations outside the Zhejiang University of Technology, metro stations, and cafés near Hangzhou Kerry center as well as a community called Emerald City to ensure the diversity of the sample data. The Zhejiang University of Technology has over 10,000 students in campus, the “Emerald City” community has nearly 8,500 households, and the Hangzhou Kerry center covers an area of 276,000 square meters and has almost 20,000 average daily passenger flows. Therefore, the area of investigation is large enough, with a potential to investigate people from different groups of society to ensure the diversity of the sample. Three investigators were separately assigned to these three different places to avoid collecting replicated questionnaires. A brief introduction of the purpose of the survey was shared with potential respondents, and they were asked whether they had used DBS services in the last month before filling in the questionnaires. If they answered “yes,” they were asked to continue answering the questions. If they said “no,” they were told to stop. Meanwhile, to increase response rates and ensure the quality of the data, potential respondents were told that they would receive a small gift after filling in the questionnaire.

Ultimately, 450 questionnaires were collected over the course of 3 months and stored in a folder. Invalid questionnaires with missing answers or duplicate answers for almost all the items were removed from the sample. Finally, 369 valid replies were ultimately obtained, and valid response rate was 82%. The size of the sample meets the requirements of the 10 times rule, which states that sample size is supposed to be larger than or equal to 10 times of structural paths in the model (Hair et al., 2016).

The demographic information of the respondents can be found in Table 1. The sample had 49.9% male respondents. As for age distribution, most of the respondents (88.7%) were between 18 and 35, which matches the population distribution of DBS users in China. Moreover, the percentage of respondents with a bachelor’s degree or above was 68.3%. More detailed information can be found in Table 1.

**TABLE 1:** Sample demographics (*N* = 369).

Characteristics	Demographic	Frequency	Percent
Gender	Male	184	49.9%
	Female	185	50.1%
Age (years)	≤18	5	1.4%
	18–25	215	58.3%
	26–35	112	30.4%
	36–45	30	8.1%
	>45	7	1.9%
Education	High school or below	60	16.3%
	College	57	15.4%
	Bachelor’s degree	145	39.3%
	Master’s degree or above	107	29%
Monthly income	Less than 2,000	79	21.4%
(RMB)	2,001–4,000	58	15.7%
	4,001–8,000	106	28.7%
	8,001–12,000	85	23.1%
	More than 12,000	41	11.1%

## Results

The article adopted SmartPLS to analyze the data for the following reasons: first, partial least squares structural equation modeling (PLS-SEM) is better for studies that focus on theory development than covariance-based structural equation modeling (SEM) (Hair et al., 2012). Second, PLS-SEM is considered to be a powerful method of analyzing a complex model with less restriction on sample size (Hair et al., 2012; Ringle et al., 2012). To predict the continuance intention of DBS users based on the extension of TAM, PLS-SEM was chosen. A two-step approach was employed to test the theoretical model by using SmartPLS version 3.0. First, we conducted an evaluation on common method bias (CMB) before all the assessments. Then, the measurement model was tested to examine the reliability and validity of the constructs. Finally, the structural model was tested to analyze the hypotheses.

### Common Method Bias

As a self-reported survey was used to collect data in this study, common method bias (CMB) may exist and threaten the reliability and validity of the results. To assess the severity of CMB, the study performed statistical analyses. First, the Harmon single-factor method (Podsakoff et al., 2003) was used to test CMB. The results showed that the single factor explains 43.44% of the total variance, which is below the threshold value of 50% (Hew et al., 2017). Second, following the procedure used by Liang et al. (2007), the study adopted the unmeasured latent method construct (ULMC) to evaluate CMB. According to the result shown in Table 2, the average of substantive variances (R_*a*_^2^) is substantially greater than average method variances (R_*b*_^2^), with a ratio of 106:1. Hence, CMB is not an issue in this study.

**TABLE 2:** Common method bias analysis.

Constructs	Indicators	Substantive variances (R_a_)	R_*a*_^2^	Method variances (R_*b*_)	R_*b*_^2^
Continuance intention (CI)	CI1	0.903	0.815	0.022	0.000
	CI2	0.914	0.835	–0.073	0.005
	CI3	0.91	0.828	0.052	0.003
Descriptive social norms (DSN)	DSN1	0.888	0.789	0.098	0.010
	DSN2	0.914	0.835	–0.043	0.002
	DSN3	0.897	0.805	–0.056	0.003
Environmental concern (EC)	EC1	0.792	0.627	–0.193	0.037
	EC2	0.852	0.726	0.106	0.011
	EC3	0.79	0.624	0.116	0.013
	EC4	0.772	0.596	–0.048	0.002
Injunctive social norms (ISN)	ISN1	0.901	0.812	0.040	0.002
	ISN2	0.889	0.790	0.058	0.003
	ISN3	0.855	0.731	–0.105	0.011
Perceived ease of use (PEU)	PEU1	0.783	0.613	0.034	0.001
	PEU2	0.857	0.734	–0.079	0.006
	PEU3	0.832	0.692	–0.009	0.000
	PEU4	0.804	0.646	0.059	0.003
Perceived usefulness (PU)	PU1	0.864	0.746	0.072	0.005
	PU2	0.898	0.806	–0.155	0.024
	PU3	0.892	0.796	0.030	0.001
	PU4	0.868	0.753	0.054	0.003
Average		0.861	0.743	–0.001	0.007

### Measurement Assessment

The measurement model was evaluated by examining internal consistency, convergent validity, and discriminant validity. Internal consistency of the constructs was tested using Cronbach’s alpha and composite reliability (CR), and values between 0.7 and 0.95 indicate a satisfactory level of reliability (Ringle et al., 2018). As shown in Table 3, the values of Cronbach’s alpha range between 0.815 and 0.903, and those for CR range between 0.875 and 0.935, thus meeting the recommended value. Convergent validity of construct means refers to what degree to two or more items of one construct is theoretically connected (Hair et al., 2016), and this parameter is examined using average variance extracted (AVE) and factor loadings (FLs). In this study, the AVE values for all the constructs ranged from 0.639 to 0.826, and were greater than the suggested benchmark value of 0.5. All the FLs of the constructs varied from 0.739 to 0.912, exceeding the recommended threshold of 0.7. Therefore, the results indicate that the measurement model had sufficient reliability and convergent validity.

**TABLE 3:** Results for reliability and validity.

Construct	Items	Factor loadings	Cronbach’sα	rho_A	CR	AVE
Perceived usefulness (PU)	PU1	0.865	0.903	0.904	0.932	0.775
	PU2	0.894				
	PU3	0.894				
	PU4	0.870				
Perceived ease of use (PEU)	PEU1	0.782	0.836	0.838	0.891	0.671
	PEU2	0.847				
	PEU3	0.830				
	PEU4	0.816				
Descriptive social norms (DSN)	DSN1	0.895	0.882	0.885	0.927	0.809
	DSN2	0.912				
	DSN3	0.891				
Injunctive social norms (ISN)	ISN1	0.906	0.857	0.866	0.913	0.777
	ISN2	0.893				
	ISN3	0.845				
Environmental concern (EC)	EC1	0.752	0.815	0.870	0.875	0.639
	EC2	0.885				
	EC3	0.812				
	EC4	0.739				
Continuance intention (CI)	CI1	0.904	0.895	0.896	0.935	0.826
	CI2	0.911				
	CI3	0.912				

Discriminant validity is the extent to which two or more constructs should not be theoretically relevant to each other (Hair et al., 2016); the square root of AVE was used to test for discriminant validity. When the AVE is larger than the correlation value of the constructs, the measurement has discriminant validity (Paulraj et al., 2008). Based on the results that are shown in Table 4, the square roots of AVE (the diagonal values) of each construct are larger than the correlation between a pair of constructs, indicating good discriminant validity. Moreover, discriminant validity was also examined with the adoption of the heterotrait-monotrait ratio of correlations (HTMT). Table 5 demonstrates that the HTMT values ranged between 0.338 and 0.776 and were all below 0.85, thus reconfirming discriminant validity. Hence, acceptable reliability, convergent validity, and discriminant validity were demonstrated in the measurement model, which meant that further testing could proceed.

**TABLE 4:** Fornell-Larcker discriminant validity.

	CUI	DSN	EC	ISN	PEU	PU
CI	0.909					
DSN	0.625	0.900				
EC	0.466	0.399	0.799			
ISN	0.583	0.676	0.439	0.882		
PEU	0.454	0.364	0.311	0.399	0.819	
PU	0.669	0.559	0.486	0.560	0.479	0.881

**TABLE 5:** Heterotrait-monotrait (HTMT) ratio.

	CUI	DSN	EC	ISN	PEU	PU
CI						
DSN	0.701					
EC	0.521	0.462				
ISN	0.661	0.776	0.516			
PEU	0.521	0.419	0.338	0.468		
PU	0.742	0.624	0.55	0.633	0.548	

### Structural Model Assessment

Before the structure model was analyzed, the study tested the collinearity of the structural model, as path coefficient would be biased if serious collinearity existed among different latent variables (Hair et al., 2012). When variance inflation factor (VIF) values of constructs do not exceed 3.3, it can be inferred that collinearity is not an issue for estimating a model (Kock, 2015). As shown in Table 6, all the VIF values of the constructs were below 3.3, which indicates that collinearity was not an issue in the estimation of path coefficient (Hair et al., 2016).

**TABLE 6:** Variance inflation factor.

Construct “CI”		Construct “PU”		Construct “PEU”	
Latent variable	VIF	Latent variable	VIF	Latent variable	VIF
DSN	2.020		1.911		
EC			1.297		1.000
ISN	2.055		2.031		
PEU	1.344		1.240		
PU	1.770				

**FIGURE 2:**
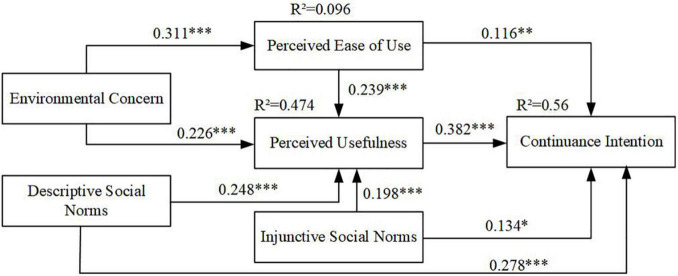
The results of the proposed model. ***p < 0.001, **p < 0.01, *p < 0.05.

After collinearity was analyzed, path analysis was conducted to verify the hypotheses. To examine the path coefficient size (β) and significance of the paths (*t* and *p* values), a non-parametric bootstrapping procedure was used to run the model with 369 collected data and 5 × 10^3^ subsamples. The results of the structural model are illustrated in Figure 2. Structural model predictive relevance was estimated using the *R*^2^ value of an endogenous latent variable. *R*^2^ values of 0.19, 0.33, and 0.7 for an endogenous latent variable can be defined as weak, moderate, and substantial, respectively, in the structural model (Chin, 1998). The proposed model accounts for 56% of variance in the continuance intention of DBS users, as shown in Figure 2. All the predictors, namely perceived usefulness (β = 0.382, *t* = 7.536, *p* < 0.001), perceived ease of use (β = 0.116, *t* = 0.627, *p* < 0.01), descriptive social norms (β = 0.278, *t* = 5.071, *p* < 0.001), and injunctive social norms (β = 0.134, *t* = 2.515, *p* < 0.05) were positively connected to the continuance intention of DBS users. Likewise, perceived ease of use, descriptive social norms, injunctive social norms, and environmental concern explained 47.4% of the variance in perceived usefulness. The paths from perceived ease of use (β = 0.239, *t* = 5.175, *p* < 0.001), descriptive social norms (β = 0.248, *t* = 4.341, *p* < 0.001), injunctive social norms (β = 0.198, *t* = 3.88, *p* < 0.001), and environmental concern (β = 0.226, *t* = 4.39, *p* < 0.001) were positively related to perceived usefulness. Meanwhile, environmental concern could also indirectly generate continuance intention toward DBS *via* perceived ease of use (β = 0.311, *t* = 5.551, *p* < 0.001). Thus, all the hypotheses were supported (see Table 7).

**TABLE 7:** Structural model results.

Hypotheses	Path	Path coefficient	*T*-value	*P*-value	Results
H1	PU- > CI	0.382	7.536***	0.000	Yes
H2	PEU- > CI	0.116	2.627**	0.009	Yes
H3	PEU- > PU	0.239	5.175***	0.000	Yes
H4	DSN- > CI	0.278	5.071***	0.000	Yes
H5	ISN- > CI	0.134	2.515*	0.012	Yes
H6	DSN- > PU	0.248	4.341***	0.000	Yes
H7	ISN- > PU	0.198	3.880***	0.000	Yes
H8	EC- > PU	0.226	4.390***	0.000	Yes
H9	EC- > PEU	0.311	5.551***	0.000	Yes

*P < 0.05, **P < 0.01, and ***P < 0.001.

## Discussion and Implications

### Theoretical Implications

Previous research studies have explored factors that affect the continuance intention of DBS users from the perspective of pro-environmental behaviors or the perspective of information system usage; however, there are very few studies that have investigated the impact of environmental concern and two-dimensional social influence on the continuance intention of DBS users. This study examined the influence of descriptive social norms, injunctive social norms, and environmental concerns on individuals’ continuance intention of DBS based on the extended TAM. Based on the analysis of the sample data of 369 users who used DBS services, the article found that all the hypotheses in the TAM were supported, confirming the results of previous studies regarding new technology continuance behaviors (Weng et al., 2017; Beldad and Hegner, 2018). Moreover, in the extended model, the *R*^2^ value of continuance intention was0.56, which is greater than the value obtained in the original model. This indicates that the extended TAM in the context of DBS continuance intention has stronger interpretation ability than the original TAM.

The results showed that perceived usefulness and perceived ease of use are the two key factors that motivate DBS user continuance intention. Perceived usefulness is the paramount predictor of DBS continuance intention, which aligns with previous studies that used the TAM (Bhattacherjee, 2001b; Weng et al., 2017). When individuals feel that DBS is improving their daily travel experience, they become more likely to continue using it; however, the effect of perceived ease of use is much lower than that of perceived usefulness (0.116 vs. 0.382). The finding is consistent with the results obtained in previous research carried out by Lin and Filieri (2015). One possible reason that explains the lower contribution of perceived ease of use on continuance intention is that people can easily use DBS with the help of a smartphone (Cheng et al., 2019). Moreover, as DBS users increase their knowledge of DBS usage, they will be familiar with using it. Hence, ease of use is losing its importance in continuance intention toward DBS. Furthermore, as was similarly demonstrated in previous studies by Weng et al. (2017), perceived ease of use has a positive and statistically significant effect on perceived usefulness, which implies that DBS being easy to use plays a significant role in creating user perception of DBS services’ usefulness in the post-use stage. Hence, enhancing DBS users’ perceived ease of use toward DBS should be considered a priority for DBS operators.

Additionally, both descriptive social norms and injunctive social norms exert a positive and significant impact on the continuance intention of DBS users. The effect of descriptive social norms on continuance intention was higher than the influence of injunctive social norms (0.278 vs. 0.134), which aligns with the findings of Beldad and Hegner (2018). These results indicate that important others’ actual use of DBS in daily life triggers users to continuously use DBS. Hence, the research uncovered salient roles of descriptive social norms and injunctive social norms in promoting the continuance intention of DBS users, and corroborates earlier findings that both descriptive social norms and injunctive social norms strongly affect an individual’s continuance intention of green products or services (Ham et al., 2015; Han and Hwang, 2017; Han and Hyun, 2018). Moreover, descriptive social norms and injunctive social norms were also found to be important predictors of perceived usefulness, which aligns with the results obtained in previous research conducted by Beldad and Hegner (2018). As a result, user perception of DBS services’ usefulness also tends to be influenced by important others, such as family members, close friends, and people who users respect. Interestingly, compared with important others’ opinions about DBS (injunctive social norms), their actual usage of this technology (descriptive social norms) exerts a higher impact on the perception of DBS services’ usefulness in daily travel. Therefore, the results demonstrate that the normative pressure from social networks exerts an important influence on DBS continuance intention and users’ perception toward DBS usefulness, which improves the understanding of the linkage between social networks and travel behavior (Chen and Deng, 2019; Hagiladi and Plaut, 2021). As was found in previous research that has examined the indirect effect of environmental concern on pro-environmental behaviors and continuance intention of green products or services (Wu J. et al., 2019; Zhang et al., 2020), this study revealed that environmental concern has a positive and statistically significant impact on the continuance intention of DBS users *via* perceived usefulness and perceived ease of use. This result indicates that users with stronger environmental consciousness are more likely to perceive DBS as a user-friendly mode of transportation and develop positive assessments for the value of DBS in daily travel. As a result, young people in China are more concerned about the environment and consider environmental protection when deciding whether to continue using an eco-friendly service (Ariffin et al., 2016). When individuals are more concerned about the environment, their perception of DBS usefulness and their perception of DBS effortless use is strengthened, which leads to increased continuance intention.

### Practical Implications

The continuous development of DBS requires public engagement and users who regularly use the service. This study offers some implications for increasing the continuance intention of DBS users that the government and DBS operators could find useful. First, the government can promote users’ perception of DBS services’ usefulness and ease of use by emphasizing the environmental benefits of this system. When individuals are more concerned about the environment, their perceptions of DBS usefulness and effortless use will be strengthened, thus increasing their continuance intention. Therefore, governments should focus on raising public awareness on environmental protection. Both traditional media and new media, such as newspapers, TV, and social media, should be used to propagate the severe consequences of environmental pollution and help citizens to understand the importance of environmental protection. Also, given the positive role of descriptive social norms and injunctive social norms in maintaining the continuance intention of DBS users, this study suggests that DBS service operators find influential reference people, such as opinion leaders and celebrities, who can express their support on the use of DBS or show their actual use through pictures or short videos on social media platforms. Another suggestion is that DBS operators should reward users who recommend DBS to others by sharing their user experience, perhaps by giving them vouchers for one week of free use. Users who sustain using DBS have the potential to promote DBS continuance usage in their social networks as well. Moreover, a suggestion might be to create a cycle-friendly climate in cities to encourage users to continue using DBS, as social interactions in urban space have an impact on the travel behavior of people (Askarizad and Safari, 2020; Li et al., 2021). Last but equally important is the significant role of perceived ease of use and perceived usefulness, which indicate that DBS operators should continue simplifying the process of using DBS services and make digital user manuals and marketing materials to increase users’ understanding of how DBS usage makes short trips more convenient.

## Conclusion and Limitations

The study proposes an extended TAM framework to explore factors that influence the continuance intention of DBS users. In summary, perceived usefulness and perceived ease of use, along with descriptive social norms, injunctive social norms, and environmental concern, are all investigated to predict how likely users are to continue using DBS systems. First, among the studied variables, perceived usefulness was found to exert the highest impact on the continuance intention of DBS users. Meanwhile, perceived ease of use was also found to have an important effect. Hence, it can be inferred that the TAM can explain the continuance intention of DBS users. Another interesting finding is that both descriptive social norms and injunctive social norms have positive impacts on the continuance intention of DBS users. These factors not only affect continuance intention directly but also have indirect impacts on continuance intention *via* perceived usefulness; however, the total effect of descriptive social norms is higher than that of injunctive social norms. This might indicate that behavioral cues from important others matter more than views that stem from strong ties in decisions on continuing to use DBS.

Given the potential environmental benefits of DBS, this study also incorporated environmental concern into the classical TAM to investigate the effect of environmental consciousness on the continuance intention of DBS users. It was found that environmental concern exerts an indirect effect on continuance intention *via* perceived usefulness and perceived ease of use. Moreover, from the perspective of total effects, environmental concern plays a larger role than perceived ease of use, so the environmental concern is a critical stimulus that affects the continuance intention of DBS users. Hence, this study confirms that individuals with a higher environmental consciousness are more inclined to continue using DBS services than those who are not eco-conscious. Therefore, the results support the idea that the extended TAM has a stronger predictive ability than the original TAM in terms of investigating continuance intention among DBS users.

Although the article conducted a comprehensive investigation, some limitations do exist. First, since the study was conducted in an eastern city in China and the sample came from a single national group, the applicability of the proposed model needs to be further validated in other countries to take into account cultural differences. Second, the explained variance of DBS continuous use intention in the study is 56%, which indicates that other factors might affect a user’s intention to continue using DBS. For instance, whether built environment factors (e.g., accessibility of the bike-sharing system) have impacts on DBS continuous use decisions. Hence, future research should investigate whether and how built environment factors affect individuals continuing the use of DBS. Finally, this study was conducted in Hangzhou, China, with a relatively small sample size. Future research should collect large samples and test the robustness of the current findings in other countries.

## Data Availability Statement

The raw data supporting the conclusions of this article will be made available by the authors, without undue reservation.

## Author Contributions

XL developed the original idea and wrote the manuscript. HL revised it critically for the important content. Both authors read and approved the final version of the manuscript.

## Conflict of Interest

The authors declare that the research was conducted in the absence of any commercial or financial relationships that could be construed as a potential conflict of interest.

## Publisher’s Note

All claims expressed in this article are solely those of the authors and do not necessarily represent those of their affiliated organizations, or those of the publisher, the editors and the reviewers. Any product that may be evaluated in this article, or claim that may be made by its manufacturer, is not guaranteed or endorsed by the publisher.

## Appendix

**APPENDIX 1:** Constructs and measurement items.

Constructs	Measurement items	Sources
Perceived usefulness (PU)	PU1: I find DBS useful in my daily life.	Davis (1989) and Bhattacherjee (2001b)
	PU2: Using DBS can improve my travel efficiency.	
	PU3: Using DBS can make my travel more convenient.	
	PU4: Using DBS can save travel time.	
Perceived ease of use (PEU)	PEU1: Interaction with DBS requires less effort.	Davis (1989), Kim et al. (2009) and Weng et al. (2017)
	PEU2: Learning to use DBS is easy for me.	
	PEU3: DBS is easy to use.	
	PEU4: It would be easy to become skillful at using DBS.	
Descriptive social norms (DSN)	DN1: My friends have participated in DBS usage.	Ru et al. (2018) and Han and Hyun (2018)
	DN2: A number of neighbors, colleagues and classmates I know have participated in DBS usage.	
	DN3: Others who are important to me have been involved in DBS usage.	
Injunctive social norms (ISN)	IN1: My family members think I should use DBS in daily life.	Ru et al. (2018) and Han and Hyun (2018)
	IN2: My close friends think I should use DBS in daily life.	
	IN3: Others who are important to me think I should use DBS.	
Environmental concern (EC)	EC1: I am concerned about the worsening environmental quality in China.	Lee (2008) and Newton et al. (2015)
	EC2: China’s environment problem is my major concern.	
	EC3: I am passionately engaged in environmental protection issues.	
	EC4: I often think about or consider how environmental quality in China can be improved.	
Continuance intention (CI)	CI1: I intend to continue using DBS in the future.	Bhattacherjee (2001b)
	CI2: I will always try to use DBS in the future.	
	CI3: I will keep using DBS as regularly as I do now.	
